# Large-scale atomistic study of plasticity in amorphous gallium oxide with ab-initio accuracy

**DOI:** 10.1038/s41598-025-93874-w

**Published:** 2025-03-19

**Authors:** Jiahui Zhang, Junlei Zhao, Jesper Byggmästar, Erkka J. Frankberg, Antti Kuronen

**Affiliations:** 1 Department of Physics, University of Helsinki, P.O. Box 43, FI-00014 Helsinki, Finland; 2 Department of Electrical and Electronic Engineering, Southern University of Science and Technology, Shenzhen, 518055 China; 3 Materials Science and Environmental Engineering Unit, Tampere University, 33720 Tampere, Finland

**Keywords:** Gallium oxide, Plasticity, Amorphous phase, Molecular dynamics, Machine learning, Glasses, Metamaterials

## Abstract

Compared to the widely investigated crystalline polymorphs of gallium oxide (${\text {Ga}_{2}\text {O}_{3}}$), knowledge about its amorphous state is very limited. With the help of a machine-learning interatomic potential, we conducted large-scale atomistic simulations to investigate the formation and plastic behavior of amorphous ${\text {Ga}_{2}\text {O}_{3}}$ (a-${\text {Ga}_{2}\text {O}_{3}}$). Amorphization of gallium oxide melt is successfully observed at ultrahigh cooling rates, including a distinct glass transition. The glass transition temperature is evaluated to range from 1234 to 1348 K at different cooling rates. Structural analysis shows similarities between a-${\text {Ga}_{2}\text {O}_{3}}$ and amorphous alumina (a-${\text {Al}_{2}\text {O}_{3}}$) in many aspects, including pair distribution function, coordination distribution, and bond angle distribution. In the tension simulations, highly plastic behavior at room temperature is observed, highly comparable to a-${\text {Al}_{2}\text {O}_{3}}$. Based on multiple quantitative characterization results, we show that a-${\text {Ga}_{2}\text {O}_{3}}$ exhibits a higher nucleation rate of localized plastic strain events compared to a-$\text {Al}_{2}\text {O}_{3}$, which can increase the material’s resistance to shear banding formation during deformation.

## Introduction

Oxide glass materials are widely used in industry and daily life because of their diverse functionalities^1,2^. However, one of the biggest limitations of their wider usage is their incapability in load bearing, despite of their extraordinary theoretical strength-to-density ratio. This is because these materials are generally considered brittle and flaw sensitive at room temperature due to the lack of an effective plastic deformation mechanism. For example, unlike crystalline materials, the dislocation mediated deformation mechanisms do not exist in oxide glass materials. Nevertheless, recent discoveries showed that some oxide glasses exhibit plasticity via diffusion based mechanisms. Amorphous silica is generally known as a brittle material, but computational studies reported that it can exhibit plasticity at room temperature if prepared under certain non-equilibrium conditions such as under high hydrostatic pressure or with ultra-fast cooling rates^3,4^. Additionally, Frankberg et al. reported that thin films of amorphous alumina (a-${\text {Al}_{2}\text {O}_{3}}$) exhibit plastic behavior under compression, tension, and shear at room temperature. Combined with computational results, they confirmed that time-dependent viscous creep is the dominant plastic deformation form and proposed an atomistic bond-switching mechanism that plays an essential role mediating the plastic deformation^5^. Microscopically, the bond switching occurs in the nucleation of localized plastic strain events (LPSEs). At lower strain rates plastic zones distribute more homogeneously in the volume, while at higher strain rates they are constrained to form shear bands^6^. Large-scale computational characterization methods are especially important for investigating plastic deformation mechanisms in amorphous materials, because small-scale methods suitable for crystalline materials, such as density functional theory (DFT) calculations, can hardly be used for non-crystalline materials due to their lack of structural periodicity. Moreover, as the lifetime of a single LPSE is some nanoseconds and includes a small amount of atoms at a time, this is usually beyond the characterization capability of short-range structural analyzing methods. Characterization methods play a crucial role in studying plastic deformation mechanisms in amorphous materials. In contrast to crystalline materials, where small-scale approaches like density functional theory (DFT) calculations are effective, the absence of structural periodicity in non-crystalline materials makes such methods largely impractical. Furthermore, since the lifetime of an individual LPSE is only a few nanoseconds and involves a limited number of atoms at any given moment, it typically exceeds the resolution of short-range structural analysis techniques. To overcome this challenge, different computational characterization methods have been proposed to provide a quantitative indication of the plasticity, such as topological constraint theory^7,8^, $D^2_\textrm{min}$ analysis^9^, ring statistics^10,11^, and coarse-grained analysis^12^. Each of them comes with advantages and disadvantages in quantitatively characterizing the plasticity and disclosing the medium-range structural information and combining different methods make the formidable challenge of quantifying. Comparing plasticity across different oxide glass materials more feasible.

Beyond the existing studies of low-temperature plasticity in amorphous silica and alumina, the current body of literature offers limited coverage on other diatomic oxide glass materials. This study is dedicated to characterize ${\text {Ga}_{2}\text {O}_{3}}$ which has analogous crystalline phases with ${\text {Al}_{2}\text {O}_{3}}$^13^. In addition, Ga^3+^ and Al^3+^ cations have distinct chemical similarity and outer electron structure and oxides of Ga and Al form solid solutions together without phase changes^14^. Due to this similarity, these two materials are often investigated together and compared with each other^15–17^. Among the known crystalline phases, the $\alpha$, $\beta$, $\gamma$, $\delta$, and $\kappa$ phases are the (meta-)stable and clearly distinguishable ones, apart from amorphous ${\text {Ga}_{2}\text {O}_{3}}$ (a-${\text {Ga}_{2}\text {O}_{3}}$). Crystalline gallium oxide is a promising semiconductor material because of its ultrawide bandgap ($\sim$4.85-5.35 eV)^18^, while a-${\text {Ga}_{2}\text {O}_{3}}$ is a novel functional material candidate for photodetectors^19–21^. Experimental studies on crystalline ${\text {Ga}_{2}\text {O}_{3}}$ have become increasingly popular in recent years, but a-${\text {Ga}_{2}\text {O}_{3}}$ has been studied only at a limited level. As a poor glass former, again similar to ${\text {Al}_{2}\text {O}_{3}}$, the synthesis of a-${\text {Ga}_{2}\text {O}_{3}}$ requires non-equilibrium conditions, such as ultrahigh cooling rates, which limits the available synthesis methods and size of the sample that can be obtained with the current technology.

From a computational perspective, DFT and ab initio molecular dynamics (AIMD) methods have been extensively used in investigating ${\text {Ga}_{2}\text {O}_{3}}$ and can provide data of high accuracy^17,22–25^. However, the high computing power cost of DFT and AIMD methods makes a larger-scale computational study of ${\text {Ga}_{2}\text {O}_{3}}$ impossible. Characterizing plasticity especially benefits from large-scale atom systems in order to avoid the occurrence of finite-size effects, as been observed when investigating other amorphous oxide materials^3,26^. Therefore molecular dynamics (MD) studies at a larger scale (more than 10^4^ atoms) become of great importance in studying plasticity in amorphous oxides^27,28^.

Machine-learning (ML) methods are emerging as important and efficient tools in interatomic potential (IAP) development^29^. From a large enough DFT calculation database, it is possible to obtain an IAP that has accuracy comparable to AIMD and is highly efficient to compute at the same time. IAPs for specific crystalline phases of ${\text {Ga}_{2}\text {O}_{3}}$ have already been developed^30,31^. Recently, a low-dimensional tabulated Gaussian approximation potential (tabGAP) aimed to provide a solution for the universal atomistic studies of ${\text {Ga}_{2}\text {O}_{3}}$ was developed^32^. With the help of this ML-IAP, it is now possible to extend the computational studies on amorphous ${\text {Ga}_{2}\text {O}_{3}}$ to larger length and time scales, avoiding finite-size effects when investigating the plastic deformation behavior. MD simulations with the ML-IAP can provide an accurate prediction regarding the mechanical behavior and mechanisms of plasticity in amorphous ${\text {Ga}_{2}\text {O}_{3}}$, providing a deeper understanding of the fundamental mechanisms and facilitating future experimental studies to validate these results.

In this work, we computationally prepared a-${\text {Ga}_{2}\text {O}_{3}}$ with the ML-IAP through a melt-quenching scheme. The obtained structure was characterized and compared with existing literature on amorphous ${\text {Ga}_{2}\text {O}_{3}}$. Next, tensile test simulations were conducted on large-scale amorphous structure with approximately one million atoms to measure the mechanical properties and investigate the plasticity of a-${\text {Ga}_{2}\text {O}_{3}}$. Plasticity observed under tension was quantitatively characterized and compared with earlier experimental and computational results on a-${\text {Ga}_{2}\text {O}_{3}}$ and on a-${\text {Al}_{2}\text {O}_{3}}$, the latter being a well-known amorphous oxide capable of significant ductility at room temperature^5^. We show that a-${\text {Ga}_{2}\text {O}_{3}}$ shows similar low temperature plasticity as a-${\text {Al}_{2}\text {O}_{3}}$, suggesting that high ductility in a-${\text {Al}_{2}\text {O}_{3}}$ is not unique but can be generalized to other amorphous oxides that fulfill the criteria introduced here to be shared between a-${\text {Al}_{2}\text {O}_{3}}$ and a-${\text {Ga}_{2}\text {O}_{3}}$.

## Results

### Amorphization and glass transition of gallium oxide

**Fig. 1:**
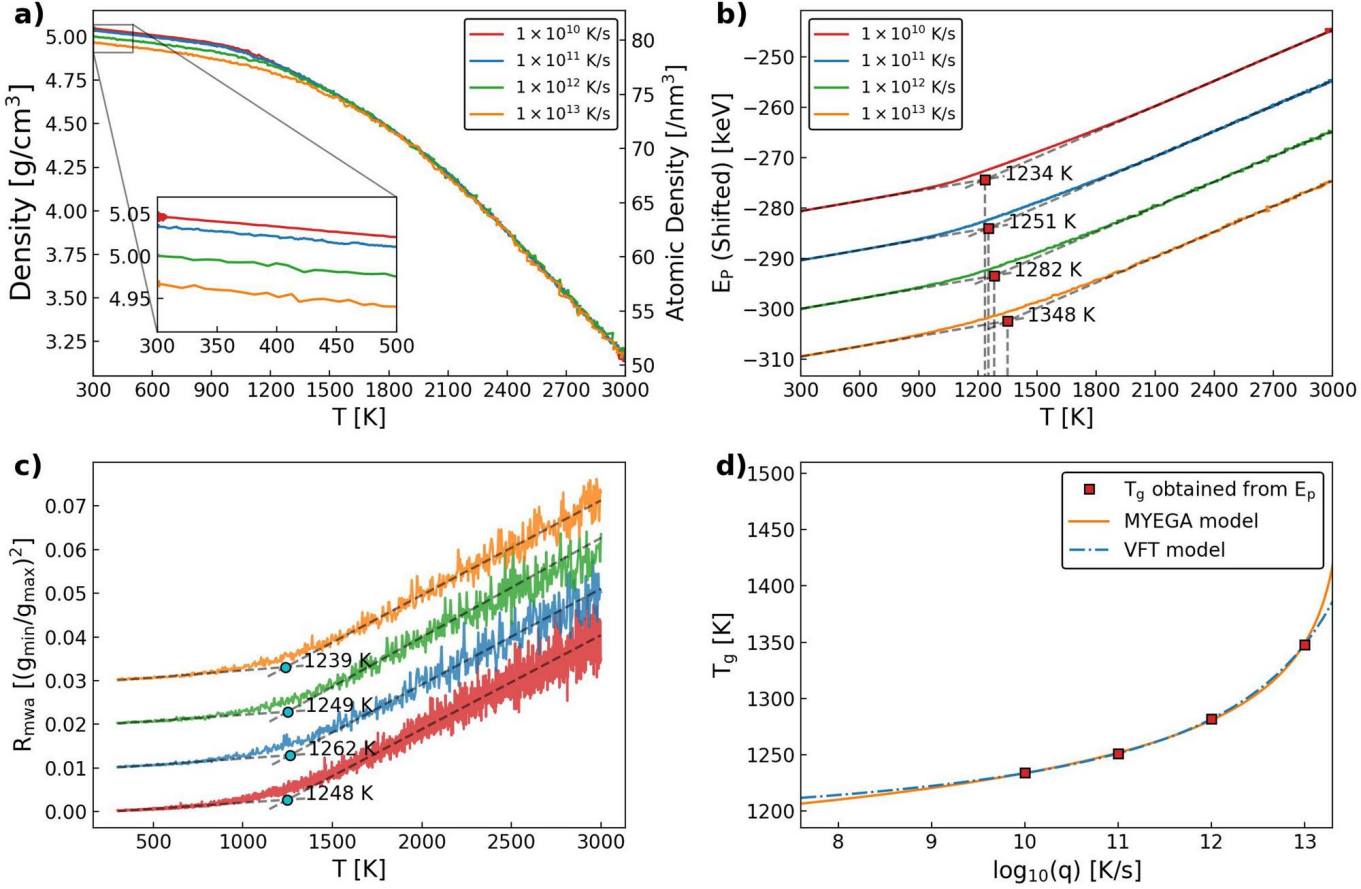
(**a**) The mass density ($\rho$) and atomic density of the ${\text {Ga}_{2}\text {O}_{3}}$ structure as a function of temperature, with different cooling rates. (**b**) Potential energy ($E_\textrm{p}$) as a function of temperature during cooling, with different cooling rates. (**c**) Modified Wendt-Abraham parameter as a function of temperature during cooling, with different cooling rates. (**d**) Fit of the $T_\textrm{g}$ data evaluation from potential energy change to the VFT and the MYEGA model.

In the melt-quenching fashion preparation of a-${\text {Ga}_{2}\text {O}_{3}}$ structures, the four cooling rates (*q*) used were $10^{10}$ K/s, $10^{11}$ K/s, $10^{12}$ K/s, and $10^{13}$ K/s. Figure 1a presents the mass density ($\rho$) and atomic density of a-${\text {Ga}_{2}\text {O}_{3}}$ as functions of temperature during quenching. At 3000 K, liquid ${\text {Ga}_{2}\text {O}_{3}}$ has a density of 3.2 g/cm^3^. Above 1800 K, density curves under different cooling rates overlap with each other. They show a linear increase with decreasing temperature. As temperature decreases below 1800 K, density increases slower and the curves diverge from each other, leading to different final densities. The highest cooling rate produced a density of 4.96 g/cm^3^, and the lowest cooling rate 5.05 g/cm^3^, with a 2% difference. At 300 K, the a-${\text {Ga}_{2}\text {O}_{3}}$ densities show approximately a 4% difference compared to recent experimental results of 4.78–4.84 g/cm^3^^16,31^, consistent with the density range reported earlier^33^. However, we also report a different density of approximately 4.25 g/cm^3^ at 2100 K, which is lower than the reported experimental value of 4.84 g/cm^3^^34^.

Regarding the atomic density, at the chosen cooling rates we obtain atomic densities ranging from 79 to 81 atoms per nm^3^ at 300 K. Results show that though a-${\text {Ga}_{2}\text {O}_{3}}$ has a significantly higher mass density due to heavier Ga atoms, its structure is not as closely packed as a-${\text {Al}_{2}\text {O}_{3}}$, which has an atomic density of approximately 97 atoms per nm^3^ at 300 K^35^.

The potential energy ($E_\textrm{p}$) as a function of temperature for all cooling rates is presented in Fig. 1b. At all cooling rates, potential energy shows a continuous and smooth decrease without an abrupt change, indicating a transition from liquid to amorphous state instead of crystallization. The evident difference of gradients at high- and low-temperature parts of the curves indicates possible glass transition during the quenching. We determined the glass transition temperature ($T_\textrm{g}$) by separately fitting the high- and low-temperature parts of the $E_\textrm{p}$-*T* curve using a linear function. $T_\textrm{g}$ is determined as the intersection point of the two fitted lines. They range from 1234 to 1348 K and show high dependence on the cooling rate. $T_\textrm{g}$ could be determined from the structural information as well, which is useful when the volume or potential energy is not obtainable. In Fig. 1c we show the values of $T_\textrm{g}$ determined from the modified Wendt-Abraham parameter ($R_\textrm{mwa}$) of the structure during cooling^36^. $T_\textrm{g}$ increases from 1239 to 1262 K, but decreases to 1248 K at q = $10^{13}$ K/s. This change is possibly a result of the greater fluctuation of the $R_\textrm{mwa}$ value during cooling, as we could see form the figure. From the calculated radial distribution function (*g*(*r*)) at each temperature, $R_\textrm{mwa}$ can be calculated as

$$\begin{aligned} R_\textrm{mwa} = \frac{g_{min}}{g_{max}}, \end{aligned}$$

where $g_{min}$ and $g_{max}$ are the minimum and maximum position of *g*(*r*) respectively.

A number of models have been proposed to describe the correlation between viscosity and temperature of glass-forming materials, from which the correlation between glass transition point and cooling rate can possibly be derived. Among them, the Vogel-Fulcher-Tammann (VFT) model is an early and widely used one, but is also believed to behave poorly for fragile glass formers^37–40^. The Mauro-Yue-Ellison-Gupta-Allan (MYEGA) model is a newer model that has been shown to behave better for poor glass formers^41^. The correlation between $T_\textrm{g}$ obtained from $E_\textrm{p}$-*T* data and *q* is based on Maxwell’s expression between relaxation time and viscosity:

1 $$\begin{aligned} \tau = \frac{\eta }{G_{\infty }}, \end{aligned}$$

where $G_{\infty }$ is the instantaneous shear modulus, and the assumption of an inverse correlation between the relaxation time and cooling rate:

2 $$\begin{aligned} \tau = \frac{T_{1}}{q} \bigg |_{T=T_\textrm{g}}, \end{aligned}$$

where $T_{1}$ is a constant. Least-squares method curve fitting is performed with the following equations and results are shown in Fig. 1d. For the VFT model, the fitting function is:

3 $$\begin{aligned} T_\textrm{g} = T_{0} - \frac{A}{\log _{10}(Bq)}, \end{aligned}$$

where *A*, *B* and $T_0$ are fitting parameters. From our data, we obtain $A=305.636$ K, $B=1.9679\times 10^{-15}$ s/K, $T_{0} = 1168.87$ K. For the MYEGA model, the fitting function is:

4 $$\begin{aligned} \log _{10}(q) = A - \frac{B}{T_\textrm{g}} \exp \left( \frac{C}{T_\textrm{g}}\right) , \end{aligned}$$

where *A*, *B* and *C* are the fitting parameters. And here we obtain $A=13.4607$ K/s, $B=7.1997\times 10^{-7}$ K^2^/s, $C=2.7763\times 10^4$ K. We see from Fig. 1d that both models can give a smooth fit to the $T_\textrm{g}$ values obtained from our simulations. The MYEGA model predicts slightly higher $T_\textrm{g}$ at $q>10^{13}$ K/s, and lower prediction of $T_\textrm{g}$ at $q<10^{10}$ K/s, compared to the VFT model. The steeper increase of predicted $T_\textrm{g}$ at high *q* region is reasonable if we consider the significant artifacts that have been observed in amorphous silica when prepared at a $q=10^{14}$ K/s^42^. It is possible that the behavior of the structure detaches quickly from a predictable behavior beyond $q>10^{14}$ K/s and such cooling rates remain out of experimental reach. At the low *q* region, verifying the performance of the model is challenging because of the occurrence of crystallization. For a-${\text {Al}_{2}\text {O}_{3}}$, crystallization has been observed at a computational cooling rate of $10^{10}$ K/s^35^. Although not observed in this computational work, a-${\text {Ga}_{2}\text {O}_{3}}$ is also assumed to require extreme cooling rates to remain amorphous, due to its reported strong tendency for crystallization^32,43^.

### Tensile test

**Fig. 2:**
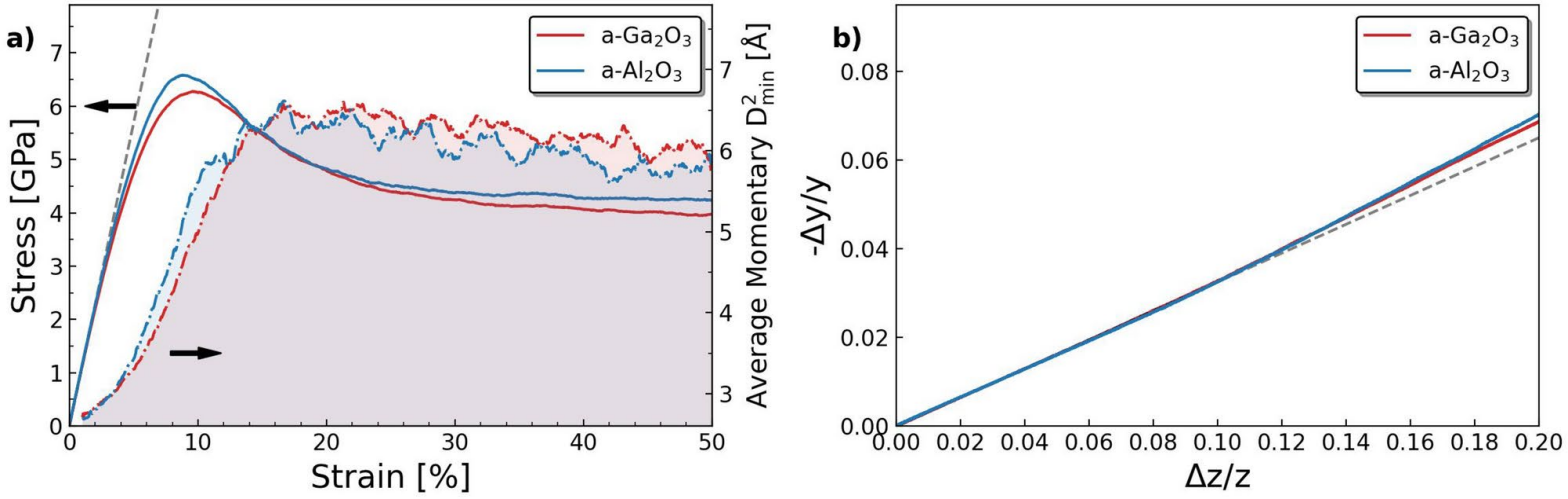
(**a**) Stress (solid lines) and average momentary ($D^2_\textrm{min}$  dashed lines) as functions of strain during tensile test. The Young’s modulus is calculated from the linear fitting of the first 1% of the curve. The $D^2_\textrm{min}$ is calculated with $r_\textrm{cut}=4.6$ Å. (**b**) Volume change on the dimension orthogonal to the strained dimension as a function of the volume change on the strained dimension in both a-${\text {Ga}_{2}\text {O}_{3}}$ and a-${\text {Al}_{2}\text {O}_{3}}$. The Poisson’s ratio of a-${\text {Ga}_{2}\text {O}_{3}}$ is fitted from the first 1% strain ($\Delta z/z <0.01$) data of a-${\text {Ga}_{2}\text {O}_{3}}$.

Tensile test simulations are performed using a large-scale amorphous structure prepared with $q=1\times 10^{12}$ K/s to avoid possible finite-size effects. The structure is elongated to 50% engineering strain at a strain rate of $5\times 10^{8}$ s^-1^. In Fig. 2a, we present the stress-strain results. Tensile test data of a-${\text {Al}_{2}\text {O}_{3}}$ structures obtained under the same conditions are also included for comparison. For both structures, the tensile stress first increases linearly with strain until the yielding. The Young’s modulus of a-${\text {Ga}_{2}\text {O}_{3}}$ is calculated by a linear fitting of the elastic deformation region of the stress-strain curve (the first 1%). For a-${\text {Ga}_{2}\text {O}_{3}}$, we obtain the Young’s modulus of $113.74\pm 0.22$ GPa. The elastic modulus evaluated from nano-indentation experiments ranges from 100 to 220 GPa^44^. Although the experimental data and simulation results are not directly comparable, we include them here as a reference. The yielding occurs later in both structures and they reach maximum stresses of 6.2 GPa for a-${\text {Ga}_{2}\text {O}_{3}}$ and 6.6 GPa for a-${\text {Al}_{2}\text {O}_{3}}$. After yielding, the stress gradually decreases and levels to a steady state flow stresses of 3.9 GPa for a-${\text {Ga}_{2}\text {O}_{3}}$ and 4.2 GPa for a-${\text {Al}_{2}\text {O}_{3}}$ measured at 50% strain. While a-${\text {Ga}_{2}\text {O}_{3}}$ has a slightly lower strength, it also shows further minor softening during the steady state flow up to 50% strain.

Figure 2a also presents the average momentary local plastic strain ($D^2_\textrm{min}$) measured during the tensile tests. Note that the $D^2_\textrm{min}$ results between a-${\text {Ga}_{2}\text {O}_{3}}$ and a-${\text {Al}_{2}\text {O}_{3}}$ are comparable here because we are able to use the same cutoff distance ($r_\textrm{cut}$) when performing the $D^2_\textrm{min}$ analysis, which will be discussed later. In comparison, we see that these two materials have similar average $D^2_\textrm{min}$ results. Atoms in a-${\text {Al}_{2}\text {O}_{3}}$ response to strain slightly faster than a-${\text {Ga}_{2}\text {O}_{3}}$ as indicated by the momentary $D^2_\textrm{min}$ value increase at a range of 2–10% strain, while a-${\text {Ga}_{2}\text {O}_{3}}$ shows a slightly higher average during the steady state flow at range of 20–50% strain. No other significant difference in plasticity can be confirmed from the average momentary $D^2_\textrm{min}$ results. To obtain a quantitative comparison of the plastic deforming ability between these two materials, further characterization is needed.

Figure 2b shows the correlation between relative deformation in directions parallel (*Z*-axis) and perpendicular (*Y*-axis) to the tensile force. Similar to the stress–strain curve, a correlation with a linear function (dashed line) between the length changes can be observed up to 10% engineering strain, while after 10% the values start to diverge from linearity. The Poisson’s ratios ($\nu$) are calculated from the 1% strain of the data. The result is 0.3253 for a-${\text {Ga}_{2}\text {O}_{3}}$, and 0.3215 for a-${\text {Al}_{2}\text {O}_{3}}$. The measured Poisson’s ratio corresponds approximately to that of metallic aluminum alloys and such high values of the ratio have been connected to increased shear deformation ability of amorphous oxide materials^5,45^. In addition, Poisson’s ratio has been reported to have the following correlation with a glass former’s fragility, which can be used to classify liquids into strong or fragile glass formers^46^:

5 $$\begin{aligned} m - 17 = 29 \left( \frac{B_{0}}{G} - 1 \right) , \end{aligned}$$

where $B_{0}$ is the bulk modulus, *G* is the shear modulus, and *m* is fragility. The correlation between $B_{0}$, *G*, and $\nu$ is formulated as:

6 $$\begin{aligned} \frac{B_{0}}{G} = \frac{2}{3} \frac{(1+\nu )}{(1-2\nu )}, \end{aligned}$$

thus the value of $B_{0}/G$ can be calculated to be 2.5287 for a-${\text {Ga}_{2}\text {O}_{3}}$, and 2.4678 for a-${\text {Al}_{2}\text {O}_{3}}$. The fragility is then calculated as 61.3328 for a-${\text {Ga}_{2}\text {O}_{3}}$ and 59.5658 for a-${\text {Al}_{2}\text {O}_{3}}$. Direct estimation of fragility is difficult because the high viscosity at temperatures near $T_\textrm{g}$ makes the time cost unaffordable. Therefore, from the data in a much broader temperature range, the fragility of a-${\text {Al}_{2}\text {O}_{3}}$ has been estimated by fitting the super-Arrhenius response in the Angell plot^47^, leading to the result of approximately 40. Therefore, both a-${\text {Ga}_{2}\text {O}_{3}}$ and a-${\text {Al}_{2}\text {O}_{3}}$ can be classified as fragile glass formers based on the simulation data.

### Structural analysis

**Fig. 3:**
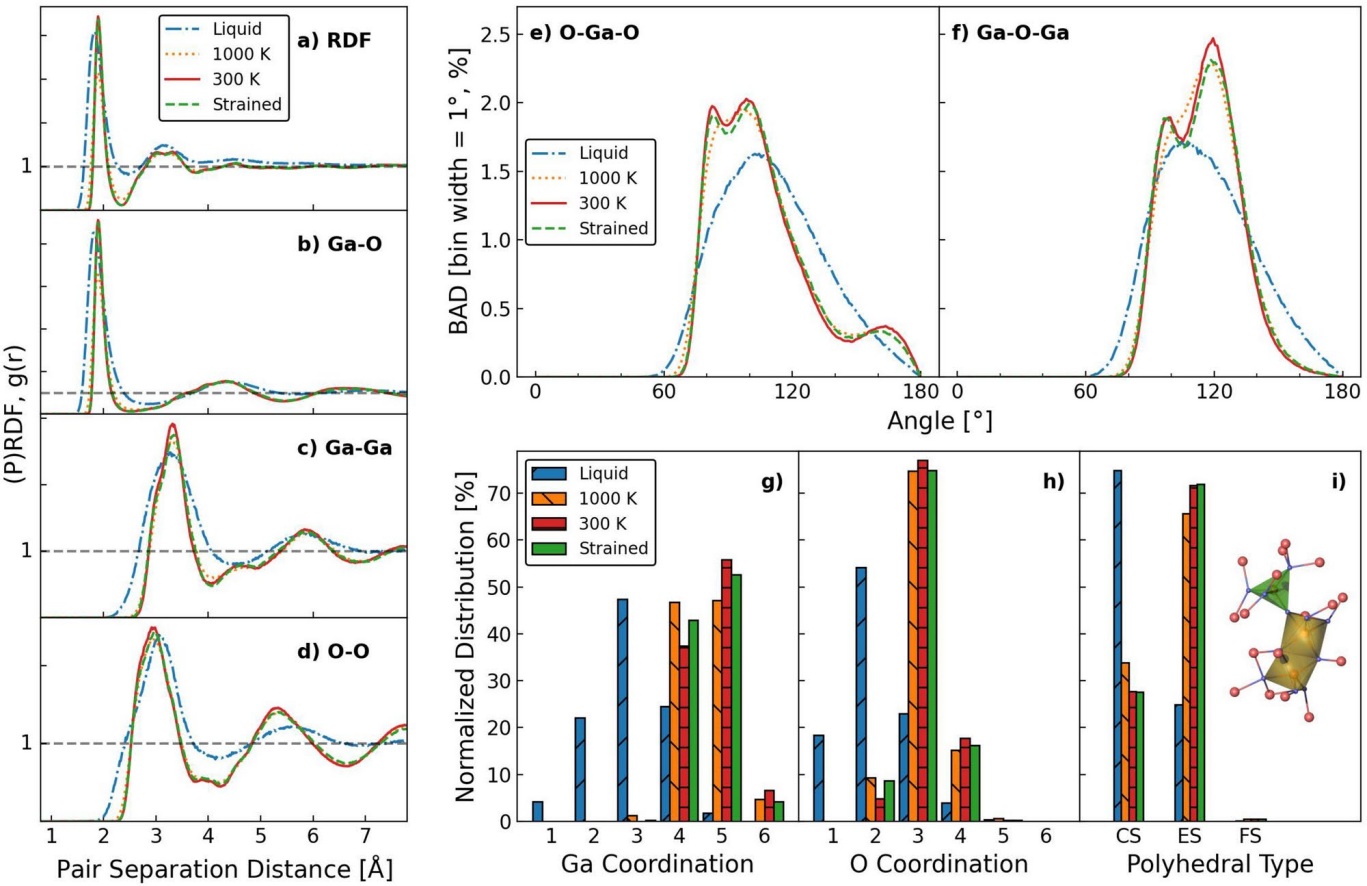
(**a**–**d**) Radial distribution function and partial radial distribution functions of the a-${\text {Ga}_{2}\text {O}_{3}}$ structures. (e, f) Bond angle distribution functions of O–Ga–O (Ga centered) and Ga–O–Ga (O centered) bond angles at 3000 K, 1000 K, 300 K and 50% strained conditions. Cutoff distance is 2.3 Å for creation of bonds. The bin size is $1^\circ$ and the results are normalized by the total bond number. (**g, h**) Coordination distributions of Ga atoms, O atoms, and (i) polyhedra type at 3000 K, 1000 K, 300 K, and 50% strained conditions. Inset shows the definition of edge-sharing (ES) and corner-sharing (CS) polyhedron in yellow and green, respectively. Gallium and oxygen atoms are colored red and blue respectively. The results are normalized by the total number of the corresponding type.

After obtaining the strained structure, a complete characterization is performed to verify that the structure prepared for tensile test simulation is amorphous, and investigate the influence of strain on the structural features. We compute the radial distribution function (RDF) and bond angle distribution (BAD) results for the four conditions of ${\text {Ga}_{2}\text {O}_{3}}$: liquid (3000 K), near the $T_\textrm{g}$ (1000 K), room temperature (unstrained, 300 K), and 50% strain (strained, 300 K). Results are shown in Fig. 3a–f. The RDF results show that for all structures there is a clear convergence to $g(r)=1$ as the pair distance increases, confirming the absence of ordering in the long range. The RDF and pairwise partial RDFs (PRDFs) of liquid ${\text {Ga}_{2}\text {O}_{3}}$ show evident differences from the other three structures (Fig. 3a–d), and much wider peaks can also be observed in the BAD plots (Fig. 3e, f). This is due to the low density and high mobility of the system at high temperature. In comparison, from 1000 to 300 K, only minor differences are observed, which confirms the occurrence of glass transition above this temperature range between 1000 and 3000 K. At 300 K, the first g(r) peak values for Ga-O, Ga-Ga, O–O pairs appear at 1.90 Å, 3.31 Å, and 2.95 Å, respectively. Although the structure is quenched without fixing the volume as constant, the RDF results are closely related to the DFT results reported earlier^31^. Comparing unstrained and strained results at 300 K, we see that strain has only a minor influence on the RDFs and PRDFs.

The BADs of the O–Ga–O and Ga–O–Ga triplets are presented in Fig. 3e, f. For liquid ${\text {Ga}_{2}\text {O}_{3}}$, both the O–Ga–O and Ga–O–Ga bond angles have a broad distribution with a single peak value around $100^\circ$. From 1000 to 300 K, the overall bond angle distribution show no change in position, but clear peaks can be seen to form at approximately $80^\circ$, $100^\circ$, and $170^\circ$ for O–Ga–O and $100^\circ$, and $120^\circ$ for Ga–O–Ga indicating an increased directionality of the bonding.

Figure 3g, h presents the coordination distribution (CD) of gallium and oxygen atoms and Fig. 3i shows the polyhedral analysis results. Similarly to the RDF and BAD results, the most significant difference can be observed in the 3000 K liquid structure, where much lower coordination numbers (such as 1-fold and 2-fold) of gallium and oxygen atoms can be observed. We see that at 300 K, 56% of the gallium atoms are 5-fold coordinated, 38% are 4-fold coordinated and 6% are 6-fold coordinated and straining at 300K has only a minor effect shifting atoms slightly from 5-fold and 6-fold coordination to 4-fold coordination. For oxygen atoms, 77% of them are 3-fold coordinated, followed by 18% 4-fold coordinated and 5% 2-fold coordinated. From 1000 to 300 K and then to strained structures, the coordination distributions show only minor differences. Honeycutt-Anderson analysis is a useful tool in metallic glasses^36,48^, it can provide useful neighbour information about the structure, as presented in Figure S2. For the analysis between the first and the second nearest neighbours, we performed the polyhedral analysis. Polyhedral analysis results in Fig. 3i show that in 300 K unstrained a-${\text {Ga}_{2}\text {O}_{3}}$, the edge-sharing (ES) polyhedra outnumber the corner-sharing (CS) polyhedra by an order of 2.7 to 1. The high fraction of ES polyhedra has been found to be correlated with the plastic deformation ability of amorphous oxide materials^12^, which partially explains why a-${\text {Ga}_{2}\text {O}_{3}}$ exhibits high ductility.

**Fig. 4:**
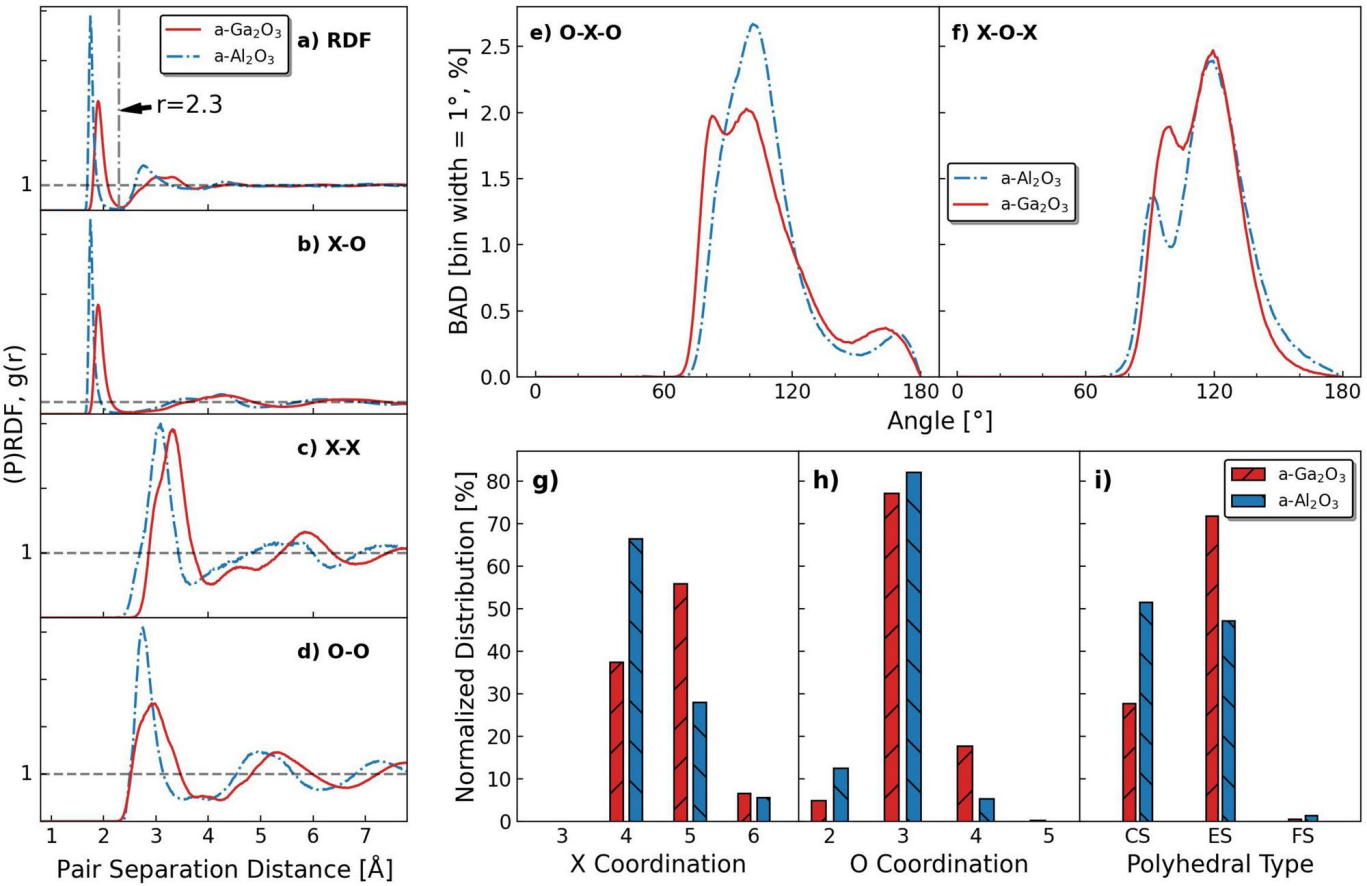
(**a**–**d**) RDF and PRDFs of the a-${\text {Ga}_{2}\text {O}_{3}}$ and a-${\text {Al}_{2}\text {O}_{3}}$ structures at 300K. (**e**, **f**) Bond angle distribution of a-${\text {Ga}_{2}\text {O}_{3}}$ and a-${\text {Al}_{2}\text {O}_{3}}$ structure. X indicates cation atom Ga or Al. Cutoff distance is 2.3 Å in creation of bonds. The bin size is $1^\circ$ and the results are normalized by the total number of bonds. (g, h) CDs of cations and O atoms, and (i) polyhedra type in a-${\text {Ga}_{2}\text {O}_{3}}$ and a-${\text {Al}_{2}\text {O}_{3}}$. Results are normalized by the total number of the corresponding type.

Comparisons of a-${\text {Ga}_{2}\text {O}_{3}}$ and a-${\text {Al}_{2}\text {O}_{3}}$ are presented in Fig. 4. Specifically, in Fig. 4a, we see that choosing 2.3 Å as the cutoff distance for the $D^2_\textrm{min}$ analysis is reasonable because it is indeed where the first minimum is for both the a-${\text {Ga}_{2}\text {O}_{3}}$ and the a-${\text {Al}_{2}\text {O}_{3}}$. However, the first peak position of the Ga-O pair is found at 1.90 Å, which is 0.15 Å larger than the first peak of Al–O pairs, indicating different cation-oxygen bond length in the two materials. This consequently leads to the lower atomic density of a-${\text {Ga}_{2}\text {O}_{3}}$ shown earlier. The first peak of a-${\text {Ga}_{2}\text {O}_{3}}$ RDF is also significantly wider in comparison, indicating more flexible bond lengths within the structure. A similar trend can be seen in the PRDFs in Fig. 4b, d as well. BAD results in Fig. 4e, f show that both types of bond angles of a-${\text {Al}_{2}\text {O}_{3}}$ are larger than that of a-${\text {Ga}_{2}\text {O}_{3}}$ in general. O–Al–O has only one peak value at $100^\circ$ instead of two present in O–Ga–O, and the first peak of the Al–O–Al distribution is slightly smaller and lower in height compared to Ga-O-Ga. Data on atomic coordination in Fig. 4g, h show that a-${\text {Ga}_{2}\text {O}_{3}}$ has a significantly higher fraction of 5-fold coordinated and a significantly lower fraction of 4-fold coordinated Ga atoms compared to a-${\text {Al}_{2}\text {O}_{3}}$. Higher coordination of Ga atoms is likely enabled by the wider distribution of allowed bond lengths shown in Fig. 4a, b. The 3-fold coordinated O atoms are similar in number, but there are also more 4-fold coordinated O atoms and fewer 2-fold coordinated O atoms in a-${\text {Ga}_{2}\text {O}_{3}}$. Figure 4i shows the medium-range polyhedral ordering, where a-${\text {Ga}_{2}\text {O}_{3}}$ has a significantly higher fraction of ES polyhedra than a-${\text {Al}_{2}\text {O}_{3}}$, indicating a good potential for low temperature plasticity in a-${\text {Ga}_{2}\text {O}_{3}}$.

### Characterization of plasticity

**Fig. 5:**
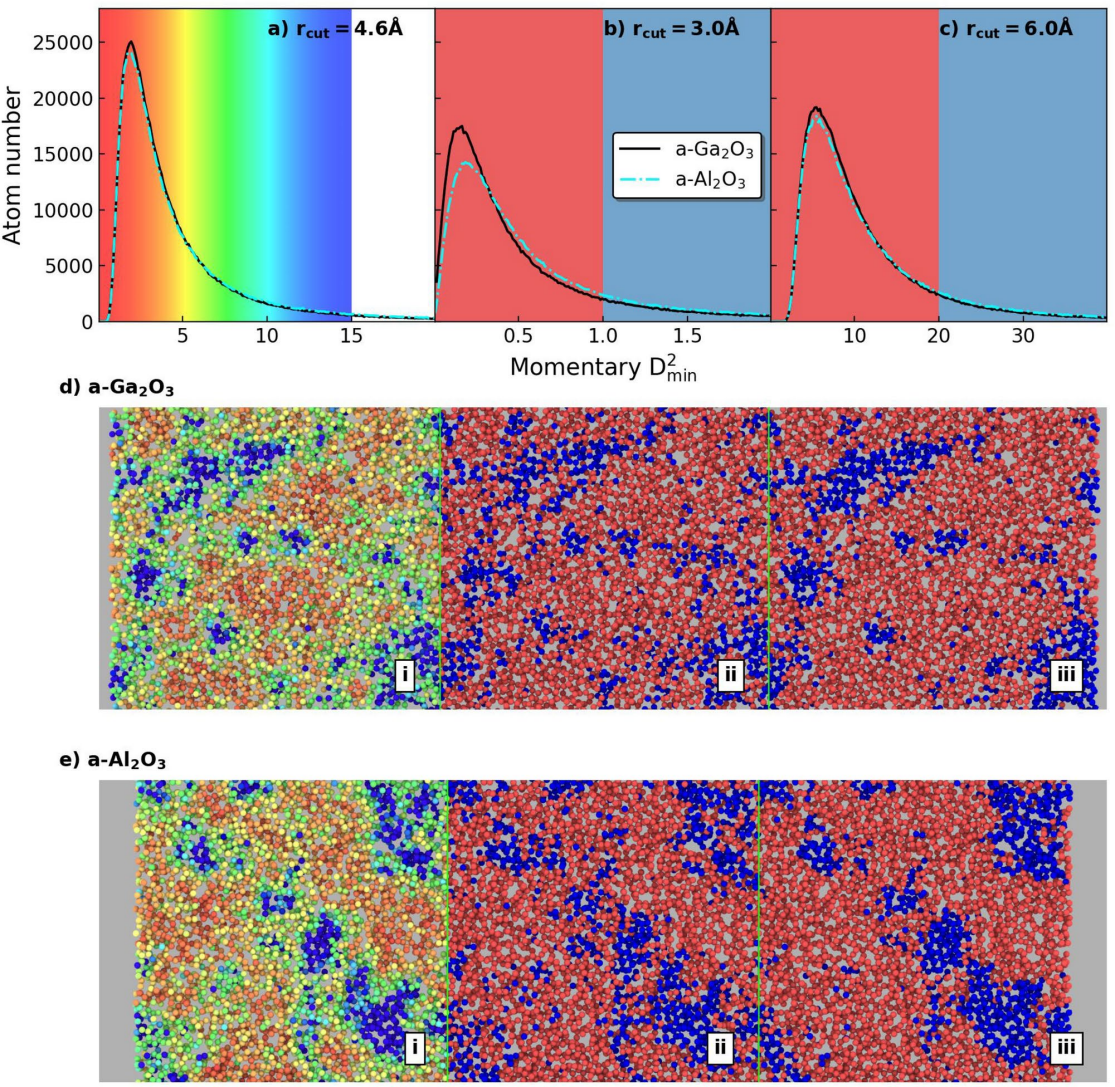
Momentary $D^2_\textrm{min}$ analysis of a-${\text {Ga}_{2}\text {O}_{3}}$ and a-${\text {Al}_{2}\text {O}_{3}}$ under 50% strain. (**a**–**c**) $D^2_\textrm{min}$ distributions calculated using different $r_\textrm{cut}$ values. (**d**–**e**) Visualizations of $D^2_\textrm{min}$ results for a-a-${\text {Ga}_{2}\text {O}_{3}}$ and a-${\text {Al}_{2}\text {O}_{3}}$, respectively. Subplots (d.i-d.iii) correspond to a-a-${\text {Ga}_{2}\text {O}_{3}}$, while (e.i-e.iii) correspond to a-${\text {Al}_{2}\text {O}_{3}}$. Each column shares the same rcut value as indicated in (**a**–**c**), and the color coding follows the background color of (**a**–**c**). The visualized cross-section is taken along the longest dimension of the structures, with a slab thickness of 5 Å. The slab width is approximately 11 Å for a-a-${\text {Ga}_{2}\text {O}_{3}}$ and 10 Å for a-${\text {Al}_{2}\text {O}_{3}}$.

Based on the tensile simulation results, local plastic strain ($D^2_\textrm{min}$) is calculated in a-${\text {Ga}_{2}\text {O}_{3}}$ and a-${\text {Al}_{2}\text {O}_{3}}$. $D^2_\textrm{min}$ is the minimum of the non-affine squared displacement as introduced by Falk and Langer^9^ and has been proven useful in capturing plastic deforming regions. In this work, we calculate momentary $D^2_\textrm{min}$, which means that the reference configuration changes with the configuration to be analyzed. A constant 1% strain difference is used between these two configurations. The stress-strain results in Fig. 2a show that the average $D^2_\textrm{min}$ results are similar for a-${\text {Ga}_{2}\text {O}_{3}}$ and a-${\text {Al}_{2}\text {O}_{3}}$ from 1 to 50% strain. This possibly indicates a similar plastic deforming ability, but we know that average $D^2_\textrm{min}$ is mainly a statistical quantity. To reveal how similar the plastic behavior is between a-${\text {Ga}_{2}\text {O}_{3}}$ and a-${\text {Al}_{2}\text {O}_{3}}$, we further analyzed the $D^2_\textrm{min}$ results from a microscopic perspective. When computing the momentary $D^2_\textrm{min}$, cutoff distance ($r_\textrm{cut}$) is an important parameter. A larger $r_\textrm{cut}$ value produce a larger $D^2_\textrm{min}$, because more atoms are included. It also helps to capture the localized deformation in longe-range and filter out the noise in short-range. In contrast, a small $r_\textrm{cut}$ value can show localized deformation that is concealed by large $r_\textrm{cut}$, but it would also produce more noise. Combining these different results, we can obtain a clearer spatial distribution of the localized deformation regions in these two materials. To probe the size of the localized deformation region in these materials, we calculate the momentary $D^2_\textrm{min}$ of both structures at 50% strain with different $r_\textrm{cut}$ values. In Fig. 5a–c, we present the momentary $D^2_\textrm{min}$ distribution with $r_\textrm{cut}=4.6$ Å, $r_\textrm{cut}=3.0$ Å, and $r_\textrm{cut}=6.0$ Å. Choosing 4.6 Å is based on the two times the first RDF minimum principle, while 3.0 Å and 6.0 Å are used for comparison between a-${\text {Ga}_{2}\text {O}_{3}}$ and a-${\text {Al}_{2}\text {O}_{3}}$. We see that different $r_\textrm{cut}$ lead to different absolute value of $D^2_\textrm{min}$ and the amount of atoms associated with the maximum peak, but the distributions are similar between a-${\text {Ga}_{2}\text {O}_{3}}$ and a-${\text {Al}_{2}\text {O}_{3}}$ at all chosen $r_\textrm{cut}$ values. Figure 5d, e presents the visualization of a cross-section taken along the long edge. A slab with 5 Å in thickness is visualized to avoid heavy overlap of atoms. Each atom is individually colored by the momentary $D^2_\textrm{min}$ value in accordance with the background color in Fig. 5a–c.

At $r_\textrm{cut}=4.6$ Å, a-${\text {Al}_{2}\text {O}_{3}}$ and a-${\text {Ga}_{2}\text {O}_{3}}$ show very similar morphology on the size of the localized deformation regions. Although a gradient color coding method in Fig. 5d helps to illustrate the transition from static to deformed regions, it makes comparison between these two materials difficult. In Fig. 5d, e, a constant threshold value ($T_c$) is used for both materials to divide the atoms into low $D^2_\textrm{min}$ ($D^2_\textrm{min}$ $< T_c$) and high $D^2_\textrm{min}$ ($D^2_\textrm{min}$ $> T_c$) groups, colored in red and blue, respectively. This helps in a direct comparison of the high $D^2_\textrm{min}$ region size between a-${\text {Ga}_{2}\text {O}_{3}}$ and a-${\text {Al}_{2}\text {O}_{3}}$. We see that when using a smaller $r_\textrm{cut}$ value, there are more but smaller regions in both systems. On the other hand, a larger $r_\textrm{cut}$ value leads to fewer but larger regions of plasticity. Comparison of the visualization results in these two materials shows a slight difference in the high $D^2_\textrm{min}$ region size. To further investigate the plastic deformation ability of these two materials, a quantitative characterization of the whole structure is performed.

**Fig. 6:**
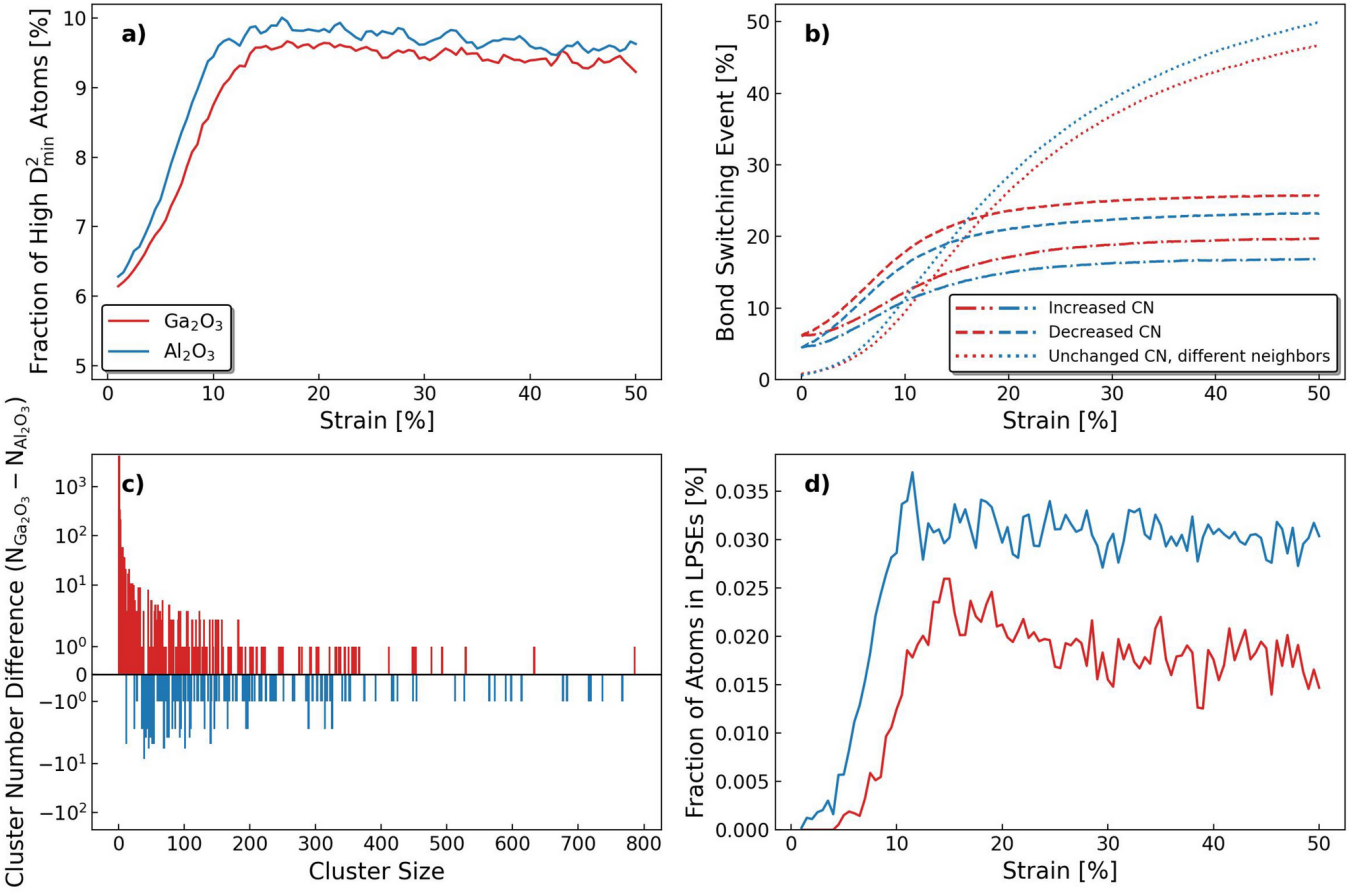
(**a**) The fraction of high $D^2_\textrm{min}$ atoms as a function of strain during the tensile test. The high $D^2_\textrm{min}$ atom here means the atoms that have $D^2_\textrm{min}$ value greater than two times the average $D^2_\textrm{min}$. (**b**) Bond change events compared to the unstrained structure. The results are classified as atoms with increased coordination numbers, decreased coordination numbers, unchanged coordination numbers but different bonded atoms. Absolute numbers are normalized according to the system size. (**c**) Difference in cluster number between a-${\text {Ga}_{2}\text {O}_{3}}$ and a-${\text {Al}_{2}\text {O}_{3}}$ at 50% strain as a function of cluster size. (**d**) The fraction of atoms in LPSEs as a function of strain during tensile test. A LPSE is here defined as a high $D^2_\textrm{min}$ atom cluster with more than 200 atoms.

As defined by Frankberg et al.^5,6^, a LPSE is a highly deformed local volume of atoms and in simulations they can be captured by the momentary $D^2_\textrm{min}$ characterization. The LPSEs play an important role during the plastic deformation of a-${\text {Al}_{2}\text {O}_{3}}$ but has not yet been applied in the simulations of a-${\text {Ga}_{2}\text {O}_{3}}$. To obtain the fraction of atoms in LPSEs in a-${\text {Ga}_{2}\text {O}_{3}}$, the $D^2_\textrm{min}$ results of the tensile test simulation are further analyzed. Although the average $D^2_\textrm{min}$ values are close, they fluctuate during the tensile test simulation. Therefore, we use a dynamic threshold value of two times the average $D^2_\textrm{min}$ ($T_d = 2\overline{D^2_\textrm{min}}$) at each moment to filter out high $D^2_\textrm{min}$ atoms ($D^2_\textrm{min}$ > $T_d$), as presented in Fig. 6. We can see in both materials that the total fraction of high $D^2_\textrm{min}$ atoms increases with strain and levels at 10% strain. In a-${\text {Al}_{2}\text {O}_{3}}$, the material’s response to strain is faster, leading to faster increase of the fraction of high $D^2_\textrm{min}$ atoms. In comparison, it reaches a maximum peak at a 2% lower strain compared to a-${\text {Ga}_{2}\text {O}_{3}}$. We notice that the point when the fraction of high $D^2_\textrm{min}$ atoms levels is later than the stress maximum, but matches the point when average momentary $D^2_\textrm{min}$ levels in the two materials, as shown in Fig. 2a. This means that the stress is associated with the rate of atoms shifting to high $D^2_\textrm{min}$ values but not with the absolute amount of them. Therefore, as they become more in the system, the rate of stress increase gradually lowers towards a negative slope. Then, the negative stress slope reverts when reaching equilibrium conditions and steady state flow. However, a slight negative slope in stress can be observed in both materials up to 50% strain, indicating a gradual and loss of material strength under tensile strain. The similar fraction of high $D^2_\textrm{min}$ atoms in both a-${\text {Ga}_{2}\text {O}_{3}}$ and a-${\text {Al}_{2}\text {O}_{3}}$ indicate that momentarily they have a very similar total amount of atoms involved in a comparable degree of localized deformation. Figure 6b presents the accumulation of bond switching events in these two materials. Compared to the unstrained structure, the atom fractions with increased and decreased coordination number (CN) have initial values of 4% for a-${\text {Al}_{2}\text {O}_{3}}$ and 6% for a-${\text {Ga}_{2}\text {O}_{3}}$, respectively. As strain increases, the fractions of increased and decreased CN first increase in the two systems, then slow down and level at approximately 20% and 26% for a-${\text {Ga}_{2}\text {O}_{3}}$, 17% and 23% for a-${\text {Al}_{2}\text {O}_{3}}$, respectively. The fraction of decreased CN is greater than that of increased CN for both materials, indicating a same degree of expansion of the systems’ volume. The fractions of unchanged CN with different neighbours starts from 0 at and quickly increase to 47% and 50% in a-${\text {Ga}_{2}\text {O}_{3}}$ and a-${\text {Al}_{2}\text {O}_{3}}$ at the end of the tensile tests, respectively. Therefore, we can say that majority of the atom translocations occur in both materials by changing neighbouring atoms while retaining the local environment. Finally, the histogram showing the size distribution of atom clusters exhibiting high $D^2_\textrm{min}$ in Fig. 6c reveals an evident difference between a-${\text {Ga}_{2}\text {O}_{3}}$ and a-${\text {Al}_{2}\text {O}_{3}}$. We see that a-${\text {Ga}_{2}\text {O}_{3}}$ has significantly more small clusters, *i.e.*, less than 20 atoms. While for a-${\text {Al}_{2}\text {O}_{3}}$, it has a much greater number of clusters in the range of 500 to 800 atoms. This shows the spatial distribution difference in these systems. Then quantitatively, results of the fraction of atoms associated with LPSEs presented in Fig. 6d show evident differences between these two materials again. Notably, a-${\text {Al}_{2}\text {O}_{3}}$ has approximately 60% higher fraction of atoms in LPSEs than a-${\text {Ga}_{2}\text {O}_{3}}$. The results indicate that although the two materials and structures have comparable amounts of highly plastic atoms, in a-${\text {Al}_{2}\text {O}_{3}}$ the LPSE regions are larger and can react to applied stress faster.

## Discussion

We conducted comprehensive computational study in this work to first verify the reliability of the ${\text {Ga}_{2}\text {O}_{3}}$ potential in amorphous state. The computational results show good consistency with existing experimental results, such as density^16,31,33,34^, and mechanical properties^44^. Comparisons are also performed with DFT data, but we note that the preparation conditions are critical for the amorphous structure, and thus we should be careful when doing such comparisons. The crystallization temperature of a-${\text {Ga}_{2}\text {O}_{3}}$ has also been reported experimentally^44,49,50^, but no crystallization was observed at the cooling rates investigated in this study. In summary, in addition to the crystalline phases validated earlier^32^, we show that the developed ML-IAP is efficient and accurate in describing the amorphous state of ${\text {Ga}_{2}\text {O}_{3}}$ as well. Oxide glasses typically exhibit low fracture toughness leading to flaw sensitivity during mechanical loading. Our simulations confirm that melt-quenching produces an a-${\text {Ga}_{2}\text {O}_{3}}$ structure sufficiently free of intrinsic geometrical defects that prevents a fracture from nucleating and propagating.

In accordance with the chemical similarity of both a-${\text {Ga}_{2}\text {O}_{3}}$ and a-${\text {Al}_{2}\text {O}_{3}}$, our simulations predict both materials to be fragile glass formers. This explains why their pure amorphous phases are difficult to synthesize with current technology and therefore they are mainly used as modifier components in glass engineering. Due to the evident similarities between a-${\text {Ga}_{2}\text {O}_{3}}$ and a-${\text {Al}_{2}\text {O}_{3}}$ and the exceptional room temperature plasticity having been reported in the latter, comparisons were made between these two materials to understand the plastic deforming ability of a-${\text {Ga}_{2}\text {O}_{3}}$ in detail.

Amorphous ${\text {Ga}_{2}\text {O}_{3}}$ showed a significantly higher glass transition temperature compared to a-${\text {Al}_{2}\text {O}_{3}}$. As shown earlier for a-${\text {Al}_{2}\text {O}_{3}}$^35^, the glass transition of a-${\text {Ga}_{2}\text {O}_{3}}$ is also dependent on the cooling rate and will dictate the mechanical properties of the obtained material. Therefore, using the same quench rate allows a better comparison between the intrinsic material properties. At a commonly shared quench rate (Fig. 2a), ${\text {Ga}_{2}\text {O}_{3}}$ glass has a slightly lower yield stress than ${\text {Al}_{2}\text {O}_{3}}$ glass. At 50% plastic strain, a-${\text {Al}_{2}\text {O}_{3}}$ is known to undergo healing towards an energetically favorable flow structure and converge towards the minimum flow stress^35^. Additionally, at 50% strain a-${\text {Ga}_{2}\text {O}_{3}}$ shows a slightly lower flow stress and a slightly higher tendency to continue softening which indicates minor changes occurring in the glassy structure up to high strains. Neither of these materials undergo hardening under plastic strain which is typical for amorphous materials due to lack of persisting plasticity mediators, such as dislocations. With currently available technology, glass transition is difficult to verify by experiments in such poor glass formers given the ultra-high quench rates involved and therefore this value is reported here also as a future reference.

We show that a-${\text {Ga}_{2}\text {O}_{3}}$ can withstand high plastic strain comparable to that characterized for a-${\text {Al}_{2}\text {O}_{3}}$, despite the interatomic potentials for the two materials being mathematically completely different (fully machine-learned versus analytical pair potential). This also serves as good cross-validation that the amorphous structures prepared for both materials are likely realistic. Building the link between structural and mechanical properties was based on one decisive observation that the same $r_\textrm{cut}$ can be used for both materials. However, although the RDFs of these two materials justify the use of this $r_\textrm{cut}$, we should still consider the approximately 8% difference in the peak positions of the cation-oxygen pair distribution indicating a significantly longer bond length for Ga-O in comparison to Al-O. In principle, such a bond length difference can lead to an atomic density difference of approximately 26%, which is close to the difference in simulated atomic densities of this work. The bond length additionally explains why the lower atomic density still does not lead to a lower average coordination number, as illustrated by the coordination distributions and polyhedral analysis results. As such structural differences will ultimately dictate the mechanical behavior, a more detailed analysis should be conducted in the future.

Although similar plastic deformation ability is observed in the macroscopic stress-strain behavior, the LPSE analysis shows distinct differences between these two oxide glasses. As a short review of how the LPSEs can reflect on the viscous creep plasticity, in Ref.^6^, it has been previously found that the number of atoms associated with the LPSEs in a-${\text {Al}_{2}\text {O}_{3}}$ remain nearly constant with increasing strain rate. At high strain rates, this leads to localization of the available LPSEs to shear bands at the location of the shear stress maximum. Accordingly, in a-${\text {Al}_{2}\text {O}_{3}}$ micropillar compression experiments at relatively high strain rates up to 10^3^ s^-1^, micropillar deformation proceeds dominantly by a slip-like shear band propagation. At low quasi-static strain rates the deformation becomes homogeneous in the whole volume with only a minor contribution of shear bands to the plasticity. Amorphous ${\text {Ga}_{2}\text {O}_{3}}$ shows a similar overall amount of high $D^2_\textrm{min}$ atoms with a-${\text {Al}_{2}\text {O}_{3}}$, however the LPSEs are smaller with fewer atoms included. This could indicate that the LPSE nucleation rate is higher in a-${\text {Ga}_{2}\text {O}_{3}}$. The nucleation rate of LPSEs was found to be a critical mediator of plasticity in oxide glasses^6^. Therefore, a possible prediction regarding the mechanical behavior of a-${\text {Ga}_{2}\text {O}_{3}}$ is that due to the higher LPSE nucleation rate, a-${\text {Ga}_{2}\text {O}_{3}}$ is predicted to yield a more homogeneous deformation at varying strain rates and to resist shear band formation more compared to a-${\text {Al}_{2}\text {O}_{3}}$. This can possibly increase the damage tolerance of a-${\text {Ga}_{2}\text {O}_{3}}$ as catastrophic shear band propagation is a known failure mechanism in amorphous materials. However, experimental verification of these predictions remain essential.

## Conclusions

In summary, we investigated the room temperature plasticity of a-${\text {Ga}_{2}\text {O}_{3}}$ with large-scale atomistic simulations based on a newly developed ML-IAP, tabGAP. Results are compared with existing experimental and computational results. On an atomistic simulation time scale, the IAP can produce an a-${\text {Ga}_{2}\text {O}_{3}}$ structure that has density and structural properties comparable to the reported properties of the material. Therefore, the new tabGAP IAP is efficient and reliable in modeling a-${\text {Ga}_{2}\text {O}_{3}}$. Computational results validate that following a melt-quenching preparation process, a glass transition occurs in ${\text {Ga}_{2}\text {O}_{3}}$.

Tensile test simulation shows that overall a-${\text {Ga}_{2}\text {O}_{3}}$ exhibits room temperature plasticity comparable to that earlier observed in a-${\text {Al}_{2}\text {O}_{3}}$ with similar number of atoms momentarily exhibiting a high local plasticity. However, differences were also found. When deformed at the same strain rate, the two materials have different fractions of atoms in the LPSE clusters that mediate the plasticity. This indicates a higher LPSE nucleation rate for a-${\text {Ga}_{2}\text {O}_{3}}$ which can increase the resistance of the material to shear banding, which is a known failure mechanism in amorphous materials. The results of this work show that the ML-IAP is a useful tool in the study of the mechanical properties of amorphous materials, providing predictive information for experimental study.

## Methods

### Preparation of the amorphous structure

Melt-quenching molecular dynamics (MD) simulations on ${\text {Ga}_{2}\text {O}_{3}}$ is performed under various cooling rates. To control the computing resource needed, a stoichiometric system with 48,000 randomly generated atoms is first heated up to 3000 K and equilibrated for 50 ps. Then the structure is cooled down to 300 K with desired cooling rate and then equilibrated for another 50 ps. Periodic boundary conditions and isothermal-isobaric (*NPT*) ensemble is applied to the system throughout the simulation. Temperature and pressure is controlled using the Nosé-Hoover algorithm^51^. Four independently prepared structures are used in the glass transition analysis. All MD simulations are performed using the LAMMPS code^52^ with the recently developed general-purpose ML-IAP (tabGAP) for ${\text {Ga}_{2}\text {O}_{3}}$^32^.

Another a-${\text {Ga}_{2}\text {O}_{3}}$ structure with significantly larger dimensions is prepared for the tensile test simulations using a cooling rate of $1\times 10^{12}$ K/s. The final size of this structure is $11.3\times 13.3\times 73.9$ nm^3^ with 960,000 atoms.

To compare the mechanical behavior, an a-${\text {Al}_{2}\text {O}_{3}}$ bulk structure with 960,000 atoms is created in a similar melt-quenching fashion as described in Ref.^53^. A Buckingham-form IAP developed by Matsui is used for a-${\text {Al}_{2}\text {O}_{3}}$ simulations^54^.

### Tensile test settings

During the tensile simulations, structures are deformed along the long axis. Periodic boundary conditions were applied to the system. The *NPT* ensemble is applied on dimensions orthogonal to the tensile deformation, the temperature and pressure is kept at 300 K and 0 bar during the stretching using the Nosé-Hoover algorithm. The simulation box is deformed every MD timestep (1 fs) without remapping the atom coordinates. The strain rate is $5\times 10^{8}$ s^-1^, and the simulation box is stretched to a maximum of 50% engineering strain. In this work, we use engineering strain and true stress in all results. The strain is defined as:

7 $$\begin{aligned} \varepsilon = \frac{L-L_{0}}{L_{0}}, \end{aligned}$$

where $L_{0}$ is the original length of the system and *L* is the length at a given strain. True stress is equivalent to the momentary tensile force divided by the cross-sectional area perpendicular to the tensile axis at the corresponding moment. Radial distribution function (RDF), bond angle distribution (BAD) and coordination distribution (CD) are analyzed using the OVITO analysis and visualization software^55^. For CD and BAD analyses, a cutoff distance of 2.3 Å is used for both a-${\text {Ga}_{2}\text {O}_{3}}$ and a-${\text {Al}_{2}\text {O}_{3}}$, which is the first minimum in the RDFs.

### Characterization of plasticity

Based on the $D^2_\textrm{min}$ analysis, in this work we define LPSE according to Ref.^6^ to make comparisons to earlier results in a-${\text {Al}_{2}\text {O}_{3}}$ meaningful. In practice, LPSE refers to an atom cluster that has more than 200 atoms that each have a $D^2_\textrm{min}$ higher than a threshold value. The threshold value equals two times the average momentary $D^2_\textrm{min}$ of the system and would dynamically change during the tensile test. To quantify the occurrence of these events, cluster analysis is performed on the atoms fulfilling the LPSE definition. The total amount of atoms included in the clusters and the size distribution of the clusters are then further compared.

The polyhedral analysis is performed in a way proposed by Zhang et al.^12^. Polyhedron in this work indicates a structure that has one center cation and multiple bonded oxygen atoms. According to the way they connect, polyhedra are classified as corner-sharing (CS), edge-sharing (ES), and face-sharing (FS).

## Supplementary Information

Supplementary Information.

## Data Availability

The datasets used and/or analysed during the current study are available from the corresponding author on reasonable request.
