# Supplementary material for: Large-scale atomistic study of plasticity in amorphous gallium oxide with ab-initio accuracy
Source: Sci Rep. 2025 Mar 19;15:9492. doi: 10.1038/s41598-025-93874-w (PMC11923202; doi:10.1038/s41598-025-93874-w)
Supplement: Supplementary file 1 — Supplementary Information. [file 41598_2025_93874_MOESM1_ESM.pdf]

Supplementary Materials

Amorphous gallium oxide structure

The structure used in tensile test simulations is shown in Figure S1. The structure is longer on the elongated dimension to avoid possible size effects.

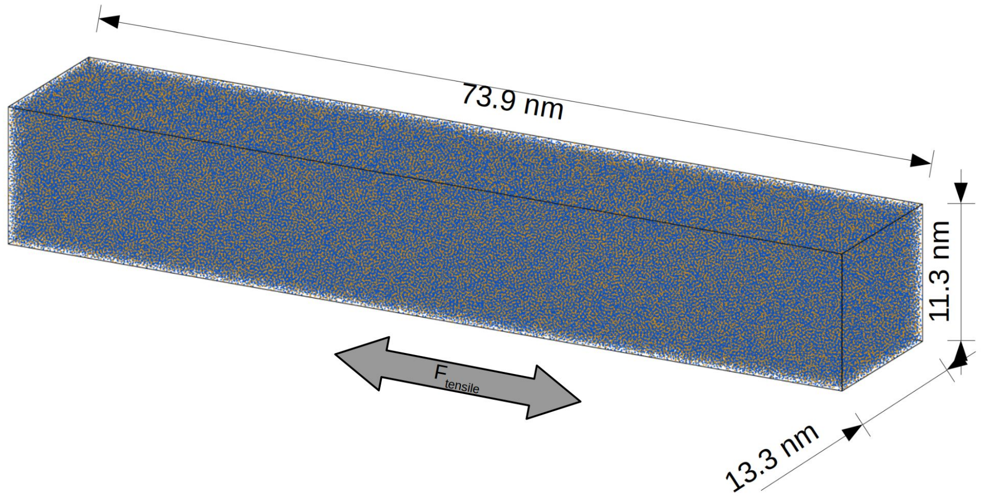

Figure S1: Amorphous structure with 960,000 atoms after melt-quenching. Gallium and oxygen atoms are colored in yellow and blue, respectively. The gray arrow indicates the direction of the tensile force, F_{tensile}.

Honeycutt-Anderson analysis during cooling

Figure S2 shows the Honeycutt-Anderson (HA) analysis results for index 1101 and 1201. Analysis is performed using self-developed tool. HA analysis is performed in such a way in this work that every pair of bonded atoms are checked, this gives the first digit of the index as 1. Then the neighbour lists of the two atoms are compared to obtain the number of shared neighbours, as second digit of the index. Then the third digit of the index can be calculated by checking the neighbour lists of the shared neighbours to see if they are bonded with each other. For the last digit of the index, it can be complex when the number of shared neighbours is large, but this is not applicable to the present cases.

The total amount of pairs in the system is around 140,000. Results show that number of both indices are pretty small, and they decrease in number during cooling. At room temperature, there are almost no either kind of the HA pair in the system. This means that for any pair of the bonded atoms, none of the neighbours of one atom appear in the neighbour list of the other, except itself. However, we notice that there is no significant difference in the results among different cooling rates. This could be due to the fact that HA analysis is still a characterization of the short-range order of the system.

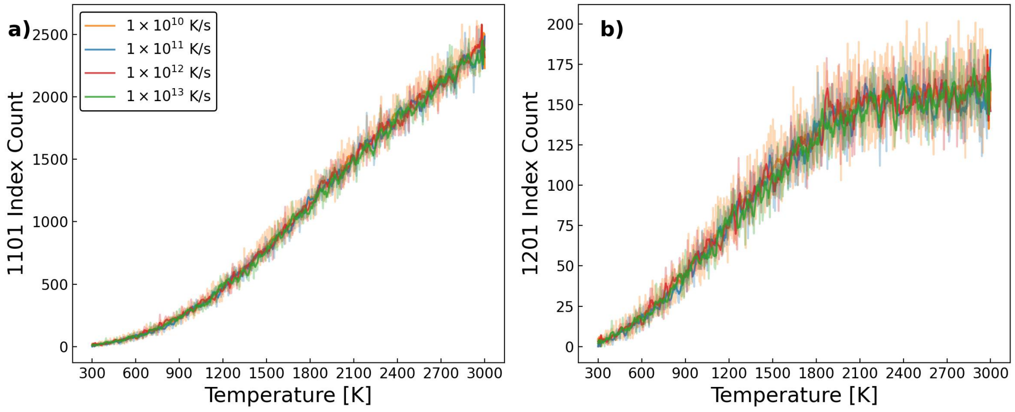

Figure S2: Honeycutt-Anderson analysis of the cooling process with different cooling rates. (a) 1101 index number. (b) 1201 index number. The semi-transparent lines are the original data and solid lines are the smoothed data for better comparison between cooling rates.

$D^2_{\rm min}$ analysis at multi-strain

Figure S3 shows momentary $D^2_{\rm min}$ distributions between the initial state, peak stress (10%) and steady-flow stress (50%). For the initial state, since $D^2_{\rm min}$ analysis needs a reference structure earlier in time, It is only possible to calculate $D^2_{\rm min}$ at 1% instead of 0%. The only different distribution is at 1% strain, which has a smaller peak position. Between peak stress and steady-flow stress, the results are pretty similar. Results also show a consistent trend on the $D^2_{\rm min}$ distribution, that it keeps broadening with strain, indicating more high $D^2_{\rm min}$ atoms at higher strain.

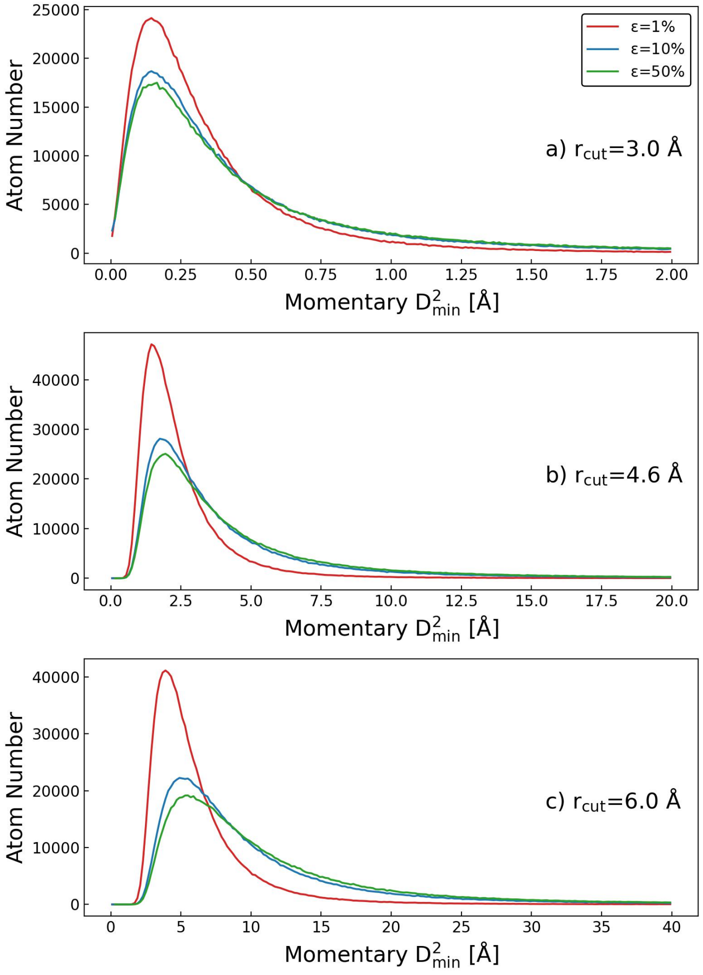

Figure S3: Momentary D^2_{min} analysis calculated at different cutoff values and strains in amorphous gallium oxide. (a) r_{cut}=3.0 Å. (b) r_{cut}=4.6 Å. (c) r_{cut}=6.0 Å.

RDF peak shift

Figure S4 shows the RDF and PRDF peaks at different temperatures and strains. The peak of Ga-O pair shows no visible shift with temperature or strain. For Ga-Ga and O-O pairs, although the maximum value positions show slight differences in the figure, they are negligible considering the error in locating the maximum value.

The reason that there is no difference on the RDF peaks could be that 1000 K is already below the glass transition temperature and the thermal expansion coefficient of Ga2O3 is rather small, at least in crystalline phases. The plastic deformation ability of amorphous Ga2O3 also helps the structure to adjust the atom positions under strain.

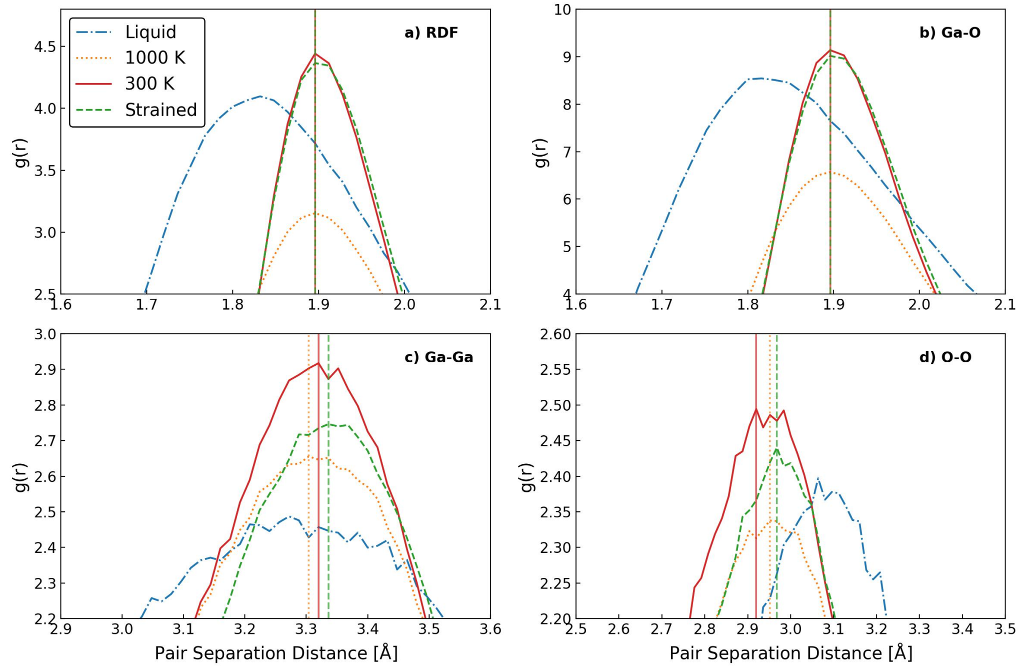

Figure S4: RDF and PRDF results at the peak postions at different temperatures and strains.
